# Supplementary figures and images for: Dysregulated Ca2+ signaling, fluid secretion, and mitochondrial function in a mouse model of early Sjögren’s disease
Source: eLife. 2024 Sep 11;13:RP97069. doi: 10.7554/eLife.97069 (PMC11390111; doi:10.7554/eLife.97069)

TMEM16a

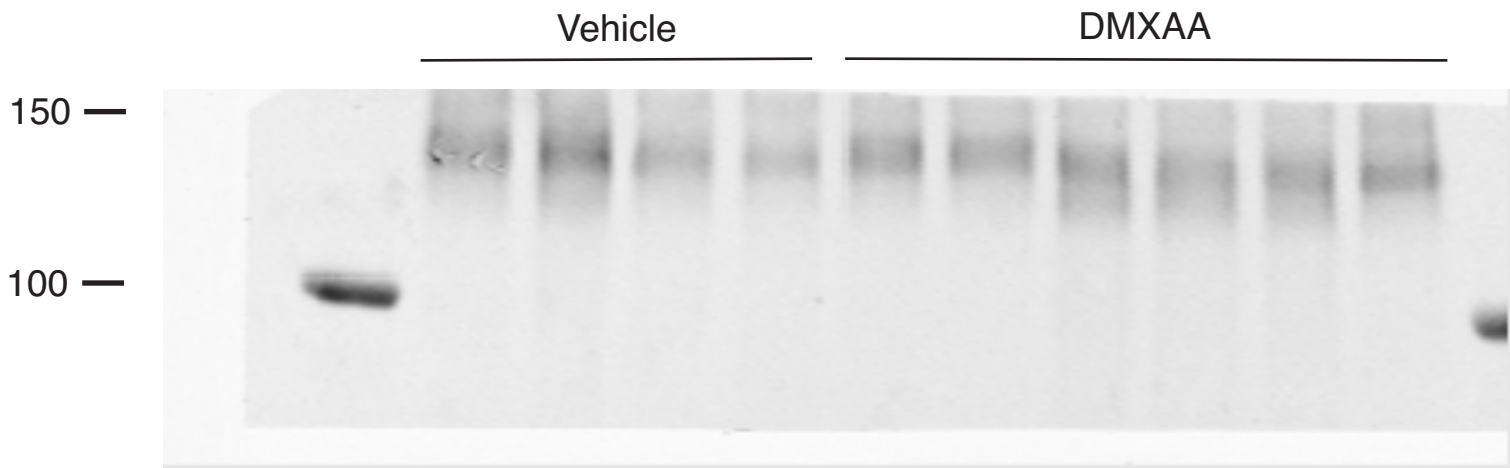

AQO5

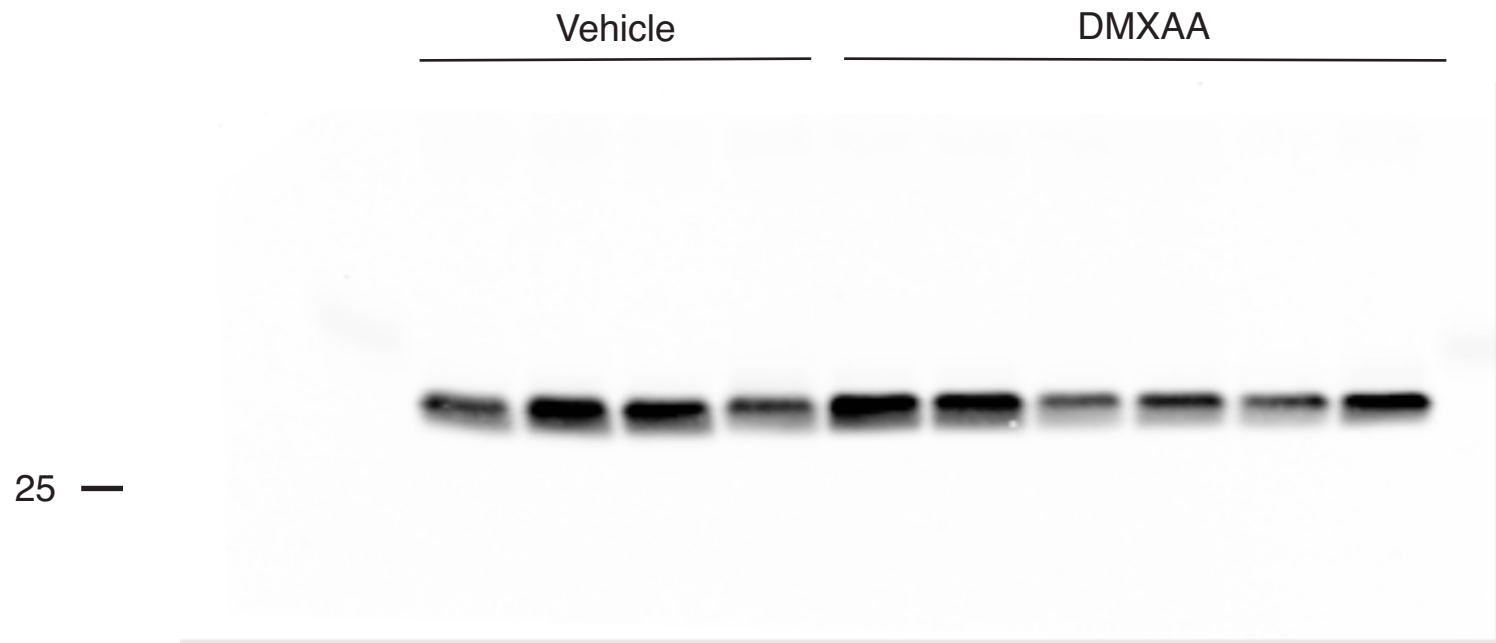

Actin

Vehicle

DMXAA

50 —  
37 —

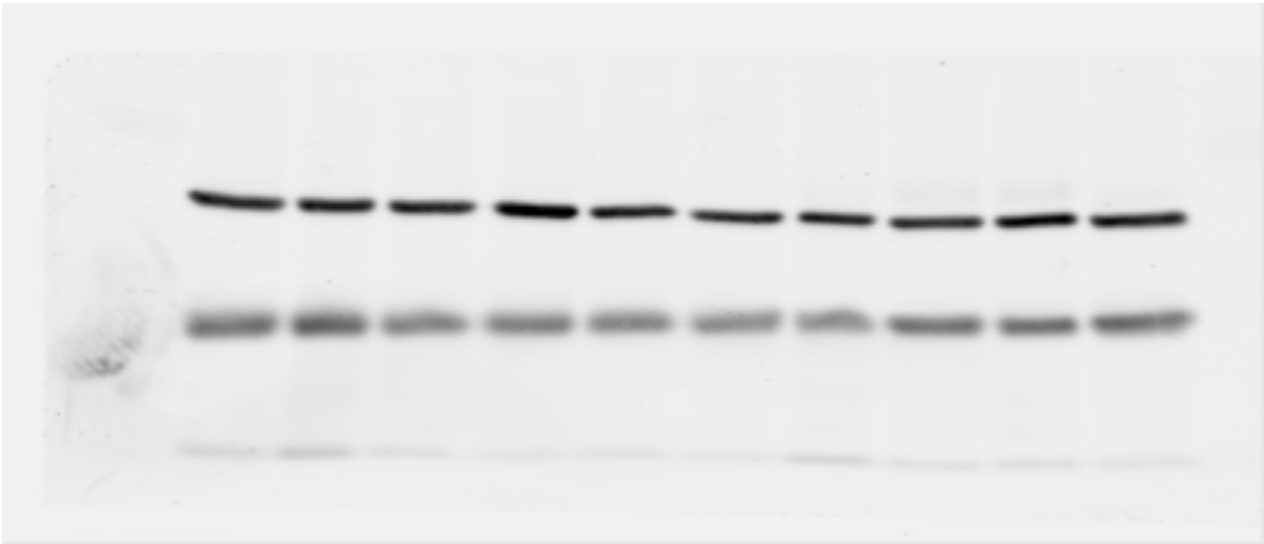

Supplement: Figure 4—source data 2. [file elife-97069-fig4-data2.zip › 97069Figure4SourceData2a.pdf]

Actin

Vehicle

DMXAA

50 —  
37 —

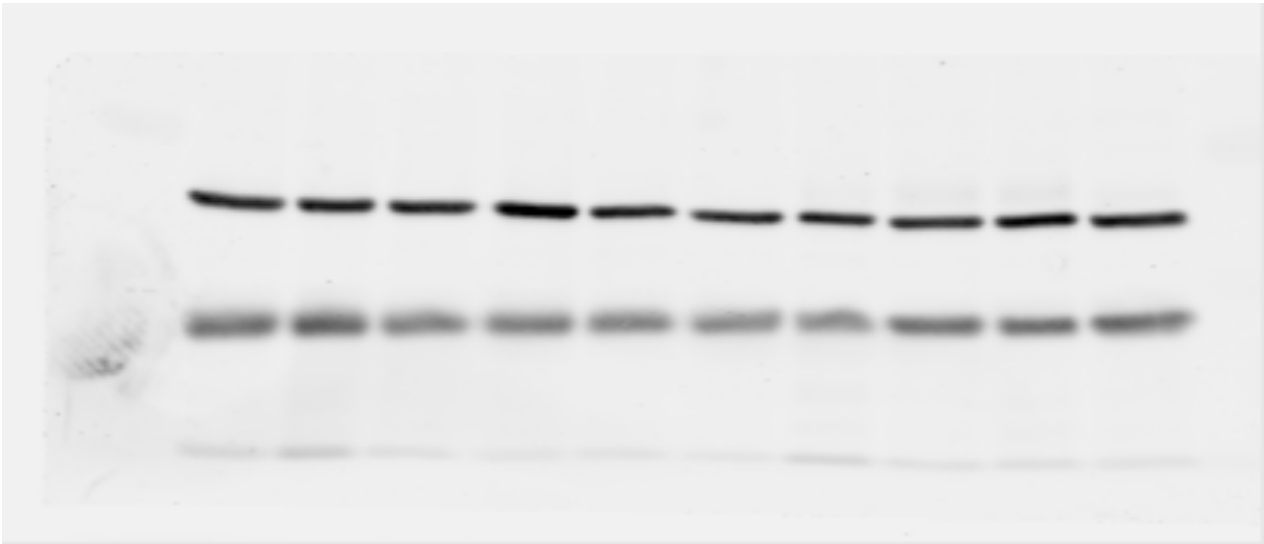

Supplement: Figure 4—source data 2. [file elife-97069-fig4-data2.zip › 97069Figure4SourceData2b.pdf]

AQP5

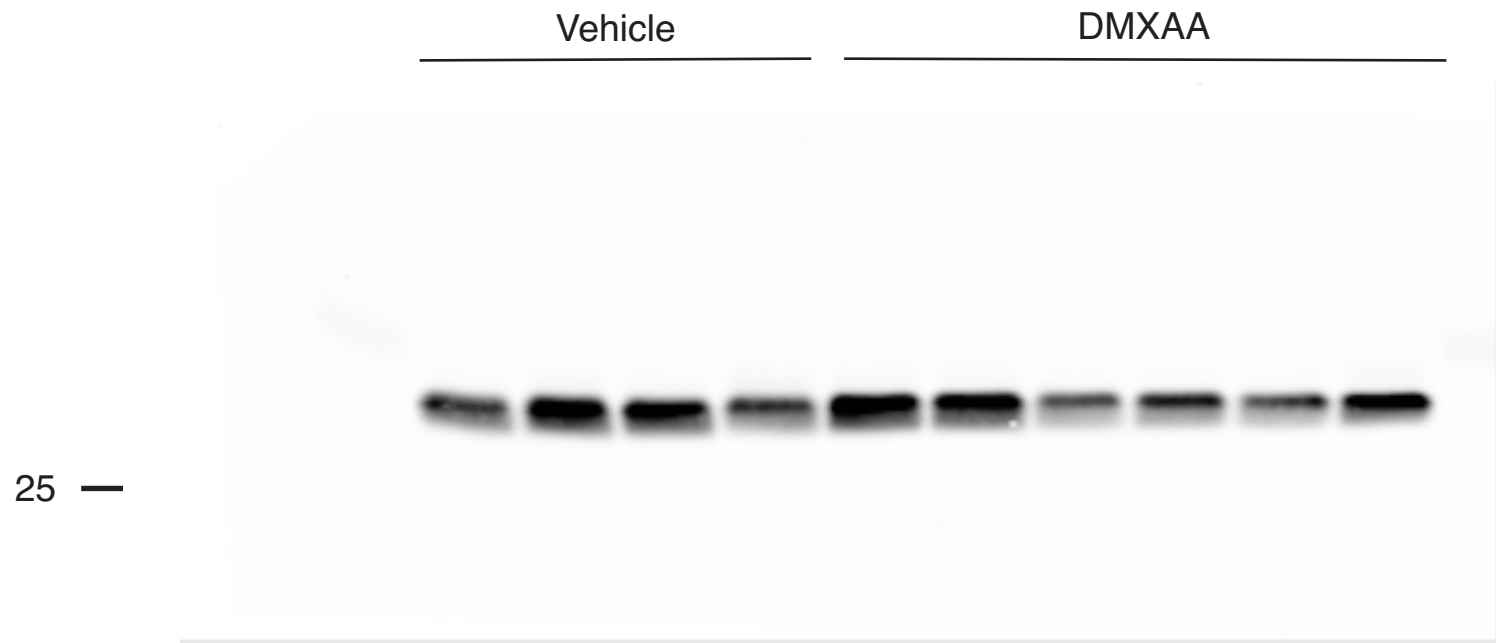

Actin

Vehicle

DMXAA

50 —  
37 —

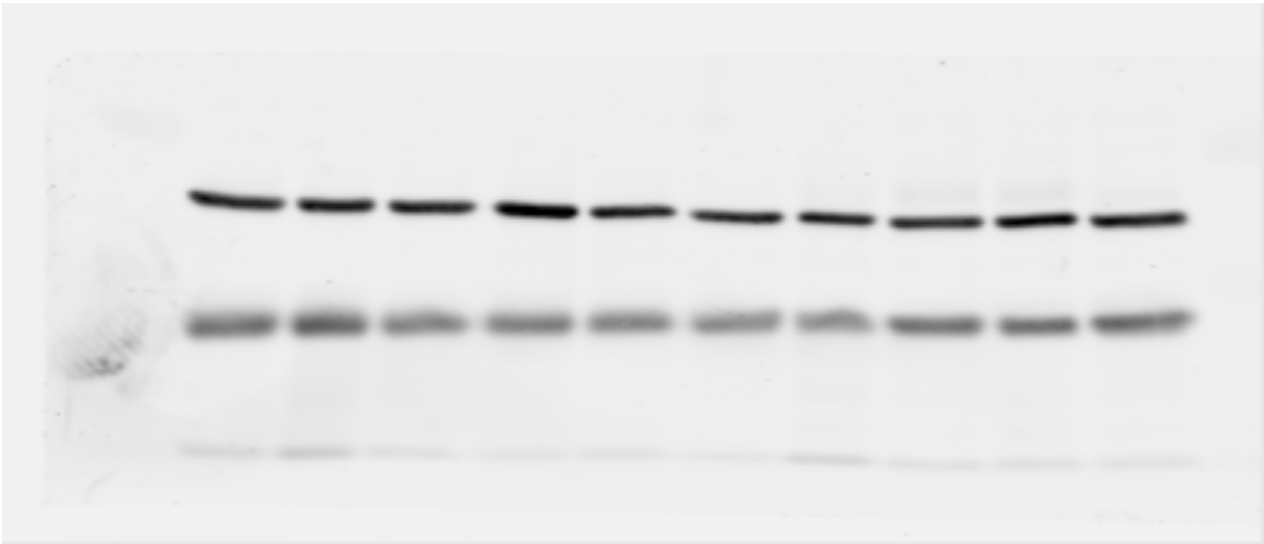

Supplement: Figure 4—figure supplement 1—source data 2. [file elife-97069-fig4-figsupp1-data2.zip › 97069Figure4FigureSupplement1SourceData2.pdf]

IP3R2

Vehicle

DMXAA

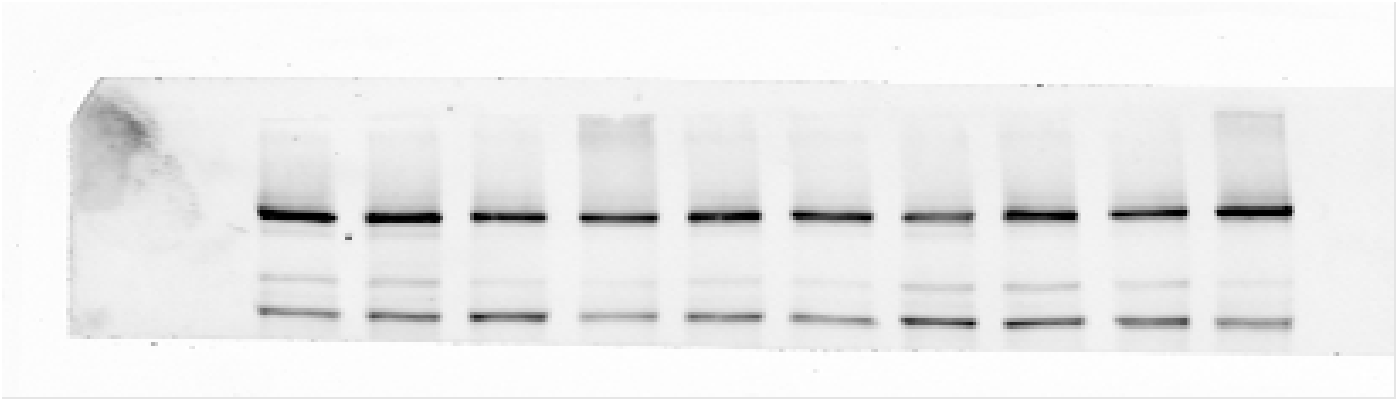

IP3R3

Vehicle

DMXAA

250 —

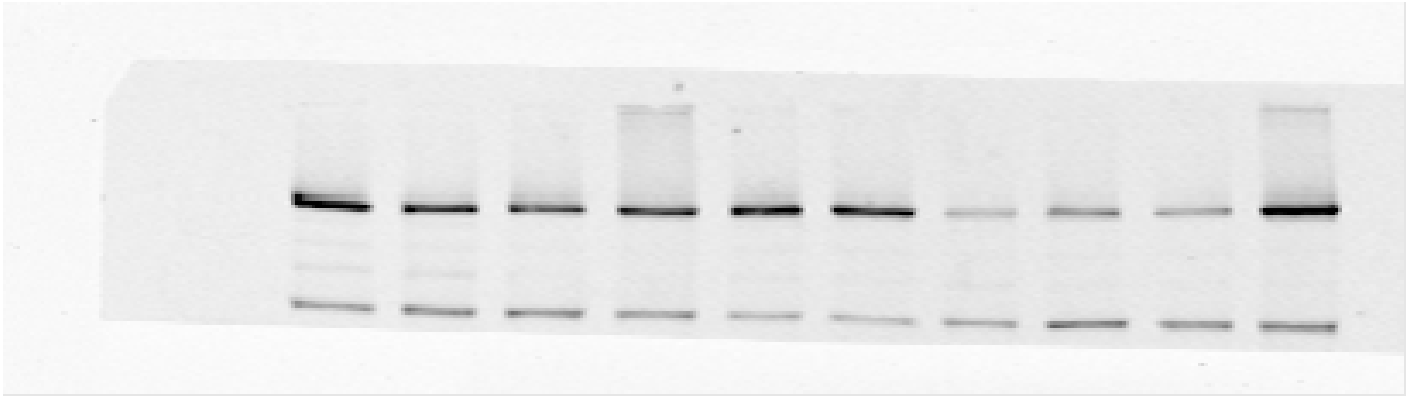

TMEM16a

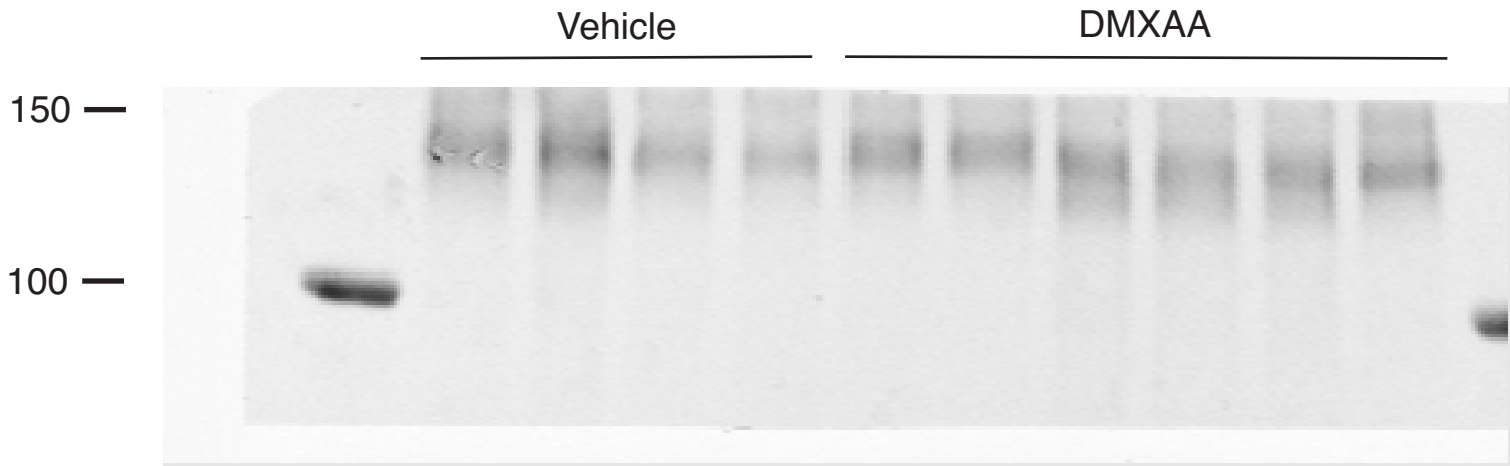

AQO5

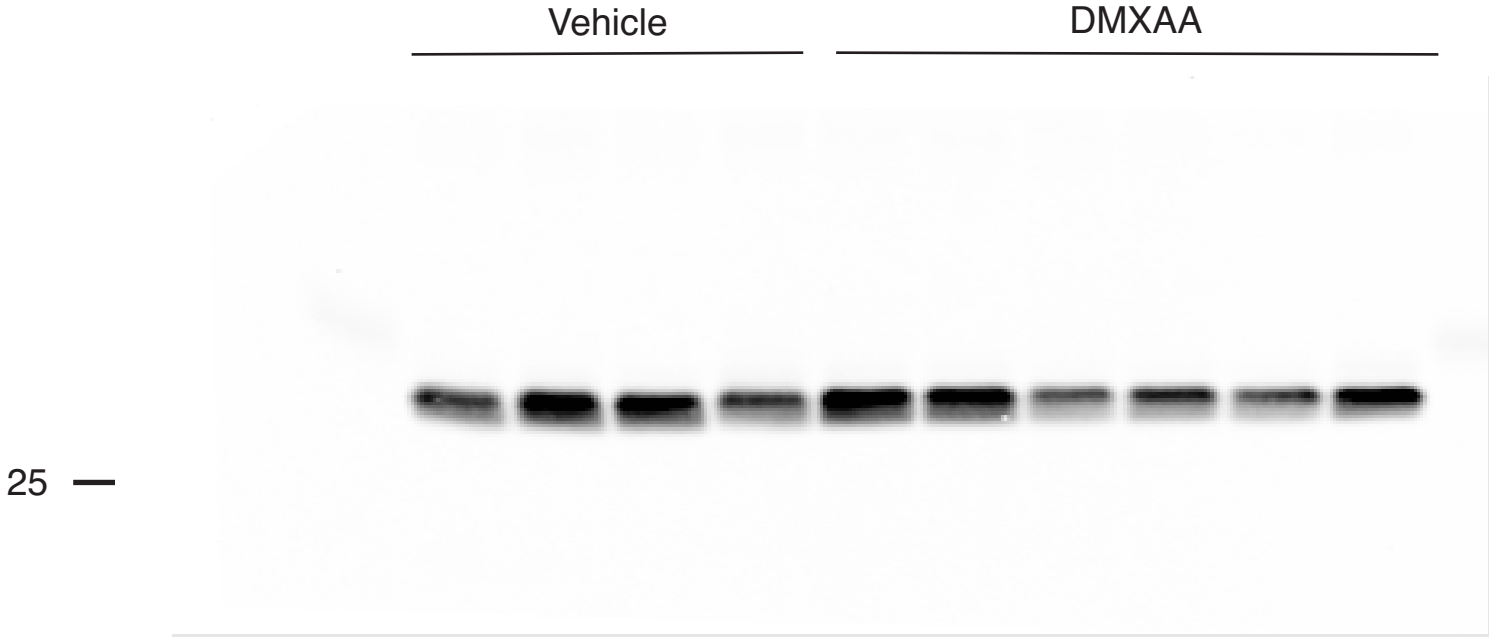

Actin

Vehicle

DMXAA

50 —

37 —

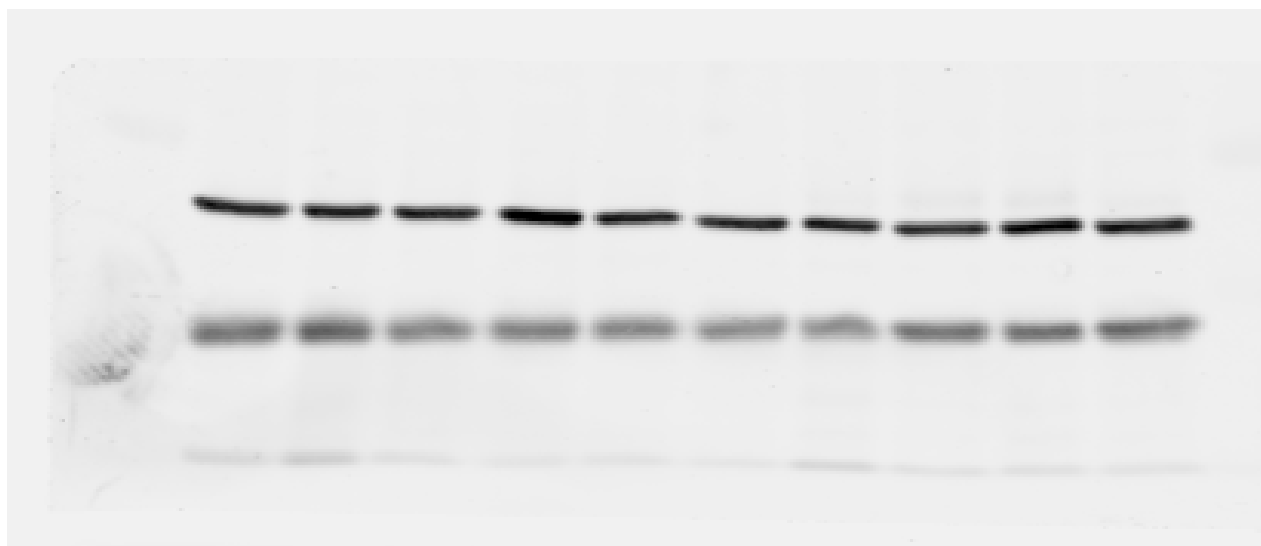

Supplement: Figure 7—figure supplement 1—source data 2. [file elife-97069-fig7-figsupp1-data2.zip › 97069Figure7FigureSupplement1SourceData2a.pdf]

IP3R3

Vehicle

DMXAA

250 —

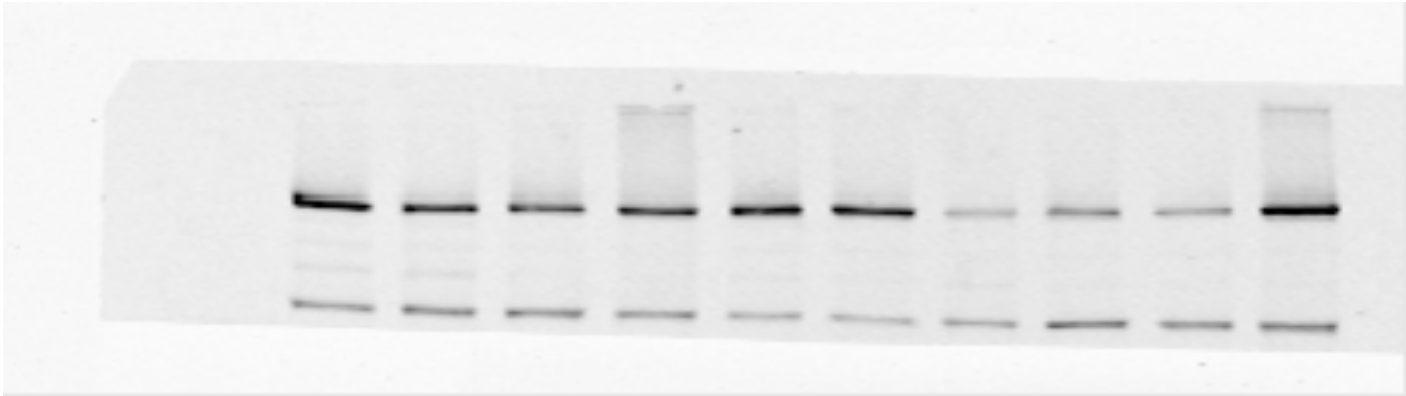

TMEM16a

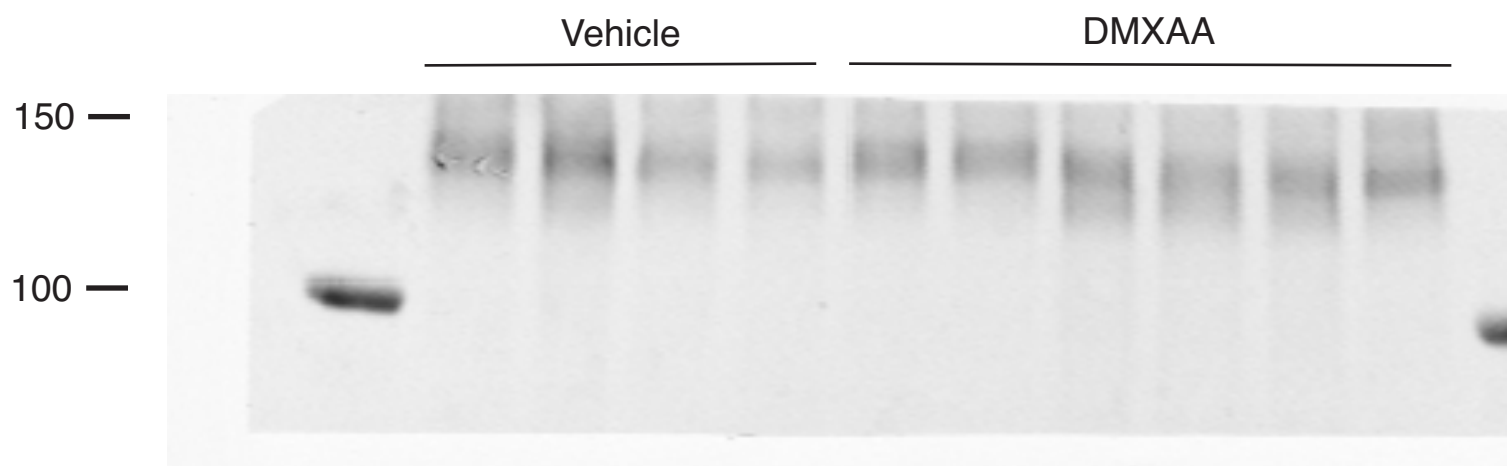

AQO5

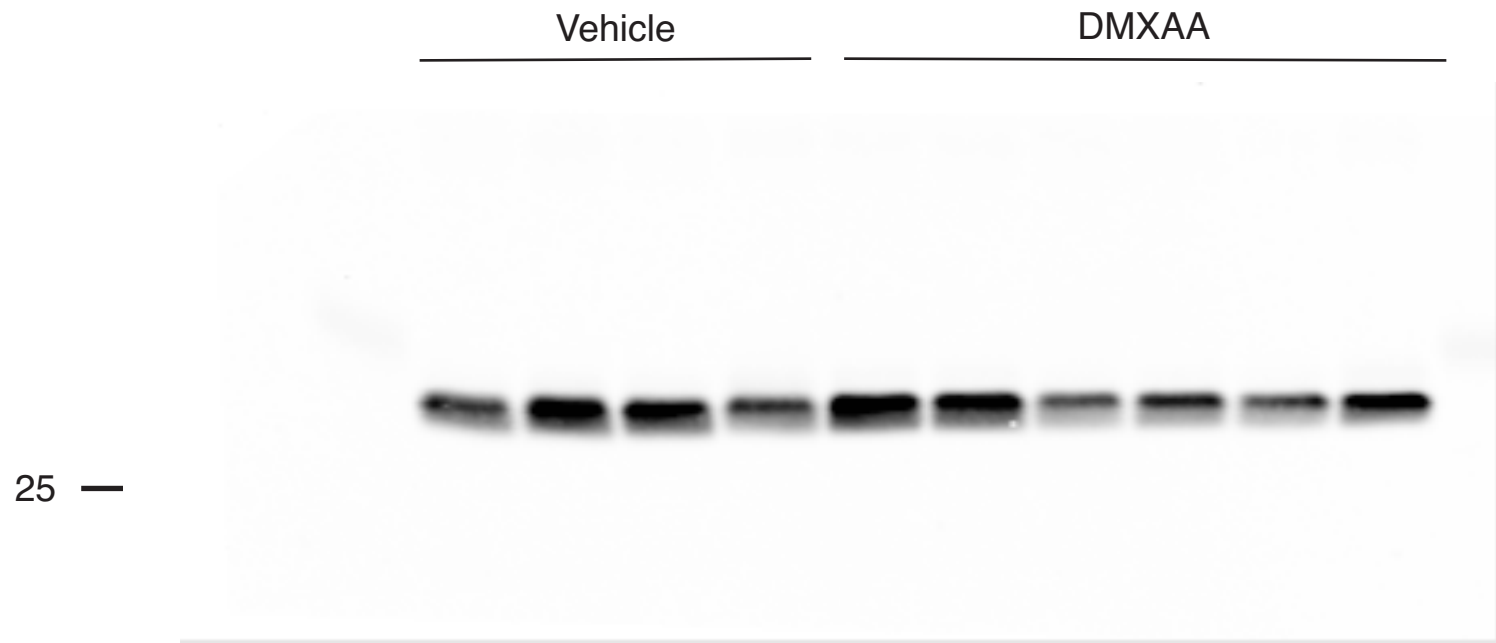

Actin

Vehicle

DMXAA

50 —

37 —

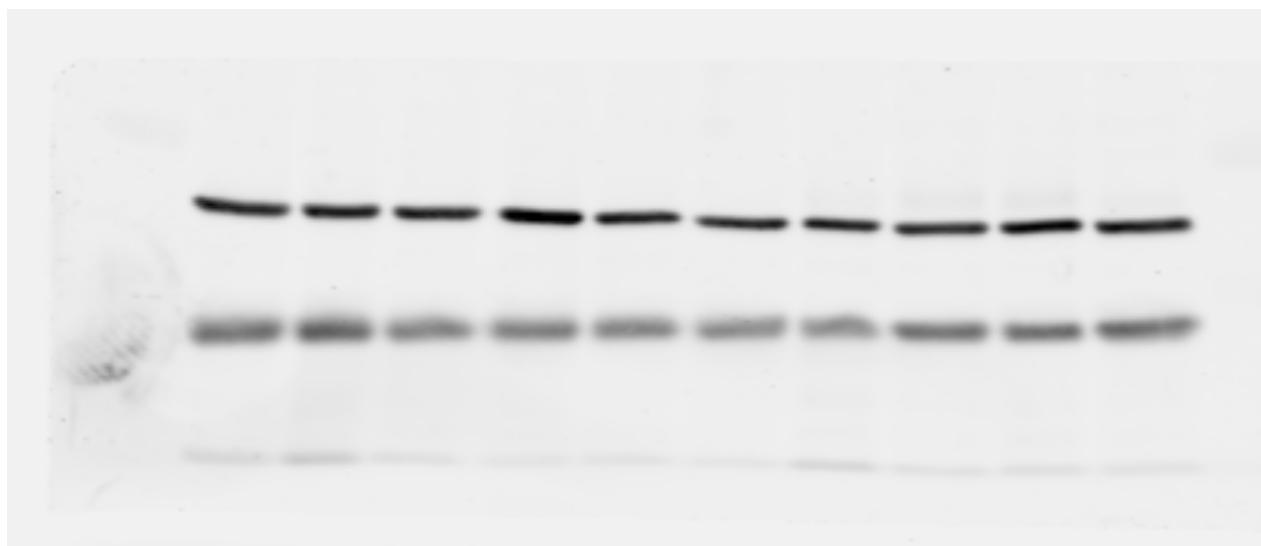

Supplement: Figure 7—figure supplement 1—source data 2. [file elife-97069-fig7-figsupp1-data2.zip › 97069Figure7FigureSupplement1SourceData2b.pdf]
